# Cell Fate Determination and the Switch from Diffuse Growth to Planar Polarity in *Arabidopsis* Root Epidermal Cells

**DOI:** 10.3389/fpls.2015.01163

**Published:** 2015-12-23

**Authors:** Daria Balcerowicz, Sébastjen Schoenaers, Kris Vissenberg

**Affiliations:** Integrated Molecular Plant Physiology Research, Department Biology, University of AntwerpAntwerpen, Belgium

**Keywords:** *Arabidopsis thaliana*, root differentiation, root hair development, initiation, planar polarity, cell fate determination, auxin

## Abstract

Plant roots fulfill important functions as they serve in water and nutrient uptake, provide anchorage of the plant body in the soil and in some species form the site of symbiotic interactions with soil-living biota. Root hairs, tubular-shaped outgrowths of specific epidermal cells, significantly increase the root’s surface area and aid in these processes. In this review we focus on the molecular mechanisms that determine the hair and non-hair cell fate of epidermal cells and that define the site on the epidermal cell where the root hair will be initiated (=planar polarity determination). In the model plant *Arabidopsis*, trichoblast and atrichoblast cell fate results from intra- and intercellular position-dependent signaling and from complex feedback loops that ultimately regulate GL2 expressing and non-expressing cells. When epidermal cells reach the end of the root expansion zone, root hair promoting transcription factors dictate the establishment of polarity within epidermal cells followed by the selection of the root hair initiation site at the more basal part of the trichoblast. Molecular players in the abovementioned processes as well as the role of phytohormones are discussed, and open areas for future experiments are identified.

## The *Arabidopsis* Root and its Developmental Zones

**FIGURE 1:**
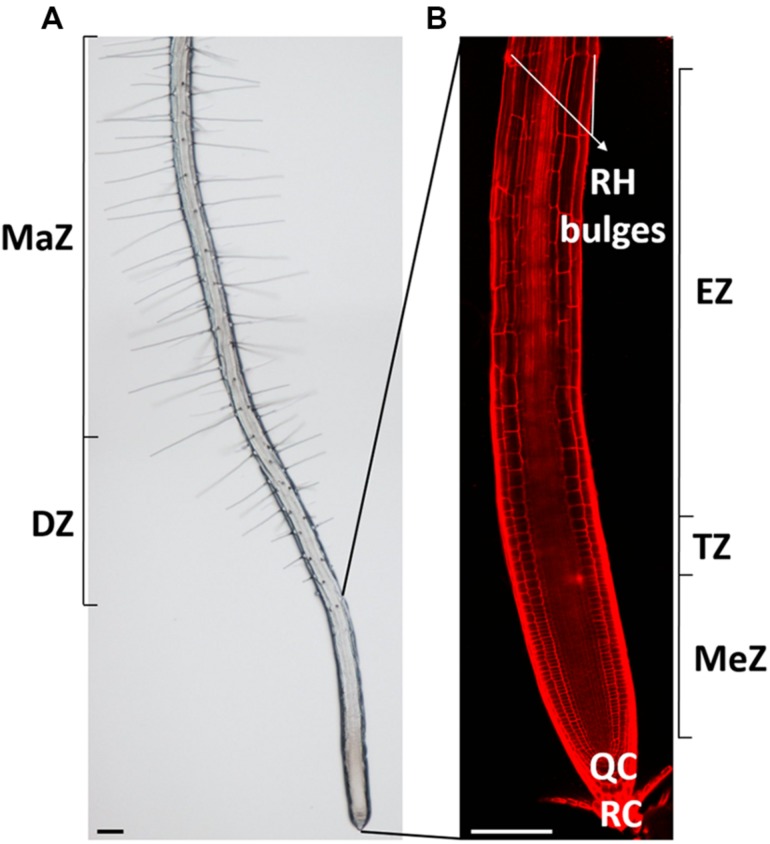
**Overview of the *Arabidopsis thaliana* root and its developmental zones. (A)** Bright-field and **(B)** confocal picture of a 7-day-old *Arabidopsis thaliana* root with its distinct developmental zones. RH, root hair; MaZ, maturation zone; DZ, differentiation zone; MeZ, meristematic zone; TZ, transition zone; EZ, elongation zone; QC, quiescent center, and RC, root cap. Scale bar = 100 μm.

Plant roots fulfill important functions and help to maximize survival and adaptation of plants to continuously changing environments. They serve in water and nutrient uptake, provide anchorage of the plant body in the soil and in some species form the site of symbiotic interactions with soil-living biota. The root body consists of many different cell types that all originate from the meristem and that all pass through consecutive phases of cellular activities before developing specialized functions and reaching maturity. The meristematic, transition, elongation, and differentiation zone of individual roots can be well defined based on their characteristic cellular activities (Verbelen et al., 2006; **Figure 1**). In the meristematic zone cells undergo mitotic divisions. As such, this zone mainly determines the root’s cell number. Upon leaving the meristem, cells modulate their physiological state and architecture to prepare for rapid elongation: they progressively develop a central vacuole, polarize their cytoskeleton and remodel their cell walls. Parallel deposition of cellulose microfibrils, transverse to the future long axis of the cell creates anisotropic cell wall mechanics that accommodate longitudinal cell expansion (Anderson et al., 2010). This highly ordered cellulose deposition by cellulose synthase enzymes is guided by cortical microtubules and related POM-POM2/Cellulose Synthase Interacting1 (CSI1) proteins (Paredez et al., 2006; Bringmann et al., 2012a,b). In the adjacent fast elongation zone, anisotropic diffuse growth, characterized by expansion of the entire cell’s surface, results in a massive increase in the cell’s volume. This process is accompanied by drastic and specific cell wall alterations, including changes in the transcription of peroxidases [which produce the reactive oxygen species (ROS) needed for cell expansion], xyloglucan endo-transglycosylase/hydrolases (XTHs) that play a role in breaking and re-joining xyloglucan cross-bridges between cellulose microfibrils, thereby weakening the cell wall (Van Sandt et al., 2007; Wilson et al., 2015) and cell wall loosening expansins (Cosgrove, 2000). Anderson et al. (2010) suggest that while the cell elongates, previously deposited cellulose fibrils reorient toward a longitudinal orientation, which might provide additional tensile strength in that dimension. In combination with rigidification of other cell wall components and changes in the protein composition, this could eventually limit longitudinal expansion so that the growth rate declines at the end of the fast elongation zone before being reduced to zero in the differentiation zone. Although epidermal wall architecture and proteins have just restrained cellular expansion in this developmental zone, root hairs yet emerge at well-defined spots of specific epidermal cells. These long, tubular-shaped outgrowths significantly increase the root’s surface area and aid in water and nutrient absorption (Keyes et al., 2013). *Arabidopsis* root hairs gained scientific attention as they represent an attractive model for studying plant cell growth and its regulation.

In the remainder of the review we will try to address the questions (1) how epidermal cells know whether or not to initiate a root hair, (2) how the position of the root hair bulge is defined, and (3) which mechanisms are required to form the actual bulge. We will further identify future challenges to even better understand the mechanism and control of root hair development.

## Definition of Root Hair Cell Fate

All cells that arise from the meristem go through the elongation zone before entering the differentiation zone. How do epidermal cells that all pass through the same developmental zones, perform similar functions, and initially look morphologically similar (despite some subtle differences, see later) gain a different cell fate?

**FIGURE 2:**
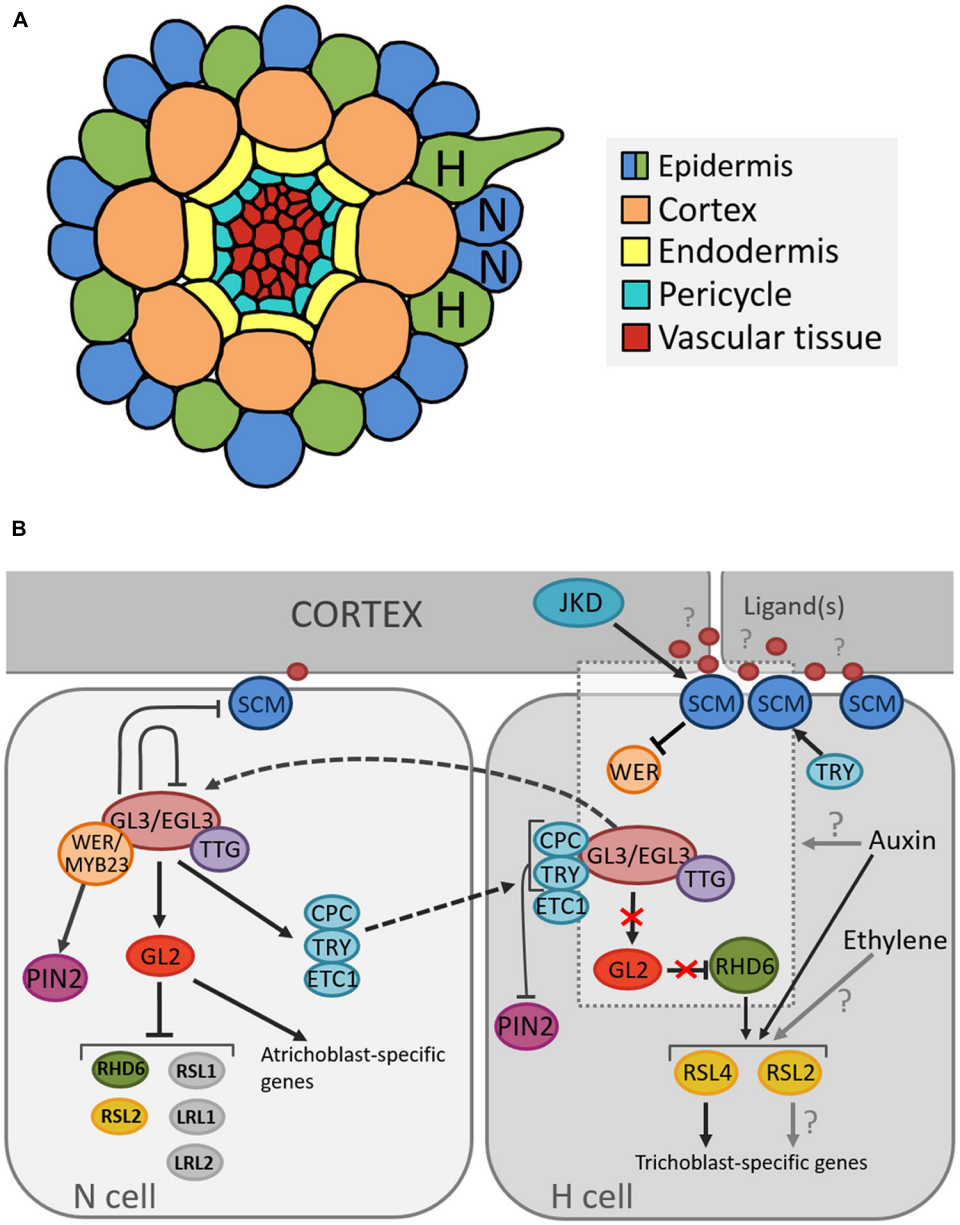
**Cellular organization of the *Arabidopsis* root and model for the position-dependent cell fate determination. (A)** Schematic representation of the cellular organization on a transverse section through the *Arabidopsis* root depicting the position of root hair (H) and non-root hair (N) cells. **(B)** Model of molecular pathways determining root hair and non-root hair cell fate in the *Arabidopsis* epidermis. Arrows, blunted lines and broken lines are indicative of positive control, negative regulation, and intra/intercellular protein movement, respectively.

In *Arabidopsis* the root epidermis is arranged in clearly distinguishable cell files arising from 16 initial cells. Upon maturation the epidermis consists of two distinct cell types: root hair cells (trichoblasts) and non-hair cells (atrichoblasts) (Dolan et al., 1993). The identity of epidermal cells is highly regulated by a position-dependent mechanism as cells that lie above the junction of two cortical cells (“H” position) produce root hairs, while cells that make contact with only one cortical cell (“N” position) remain hairless. Since the cortex is a ring of eight cells, the trichoblasts are always arranged into eight files and trichoblast cell files rarely directly neighbor each other (Dolan et al., 1994; **Figure 2A**).

The patterning of both epidermal cell types is first established during the early stages of embryogenesis, which is far before the first signs of epidermal differentiation. The expression of GLABRA2, a transcription factor required for specification of the atrichoblast cell fate, can already be detected in the heart stage embryo (Lin and Schiefelbein, 2001). In addition to genetic pathway differences, a cellular dimorphism between trichoblasts and atrichoblasts already exists in the meristematic zone, long before actual root hair formation (Dolan et al., 1994): root hair cells are shorter (Dolan et al., 1994) and less vacuolated (Galway et al., 1994), they have a denser cytoplasm (Dolan et al., 1994) and display a higher cell division rate (Berger et al., 1998).

Several genes that regulate cell fate determination and cause these subtle differences have been described (**Figure 2B**). Many studies suggest that the root hair cell fate depends on the absence of an inhibitory pathway that prevents differentiation into a hair cell (Wada et al., 1997; Lee and Schiefelbein, 1999). One of the key components in this network is TRANSPARENT TESTA GLABRA (TTG1), which encodes a small protein with WD40 repeats involved in creating protein-protein interactions (Walker et al., 1999). TTG1 forms a complex with the basic Helix-Loop-Helix (bHLH) transcription factors GLABRA3 (GL3) and ENHANCER OF GLABRA3 (EGL3) (Payne et al., 2000; Esch et al., 2003; Zhang et al., 2003). As loss-of-function mutations in each of these genes result in excessive trichoblast production, they were found to inhibit the root hair cell fate (Galway et al., 1994; Bernhardt et al., 2003; Zhang et al., 2003).

The activity of the GL3/EGL3/TTG1 complex depends on the relative abundance of two MYB transcription factors, WEREWOLF (WER) and CAPRICE (CPC) that act in opposition to each other (Wada et al., 1997; Lee and Schiefelbein, 1999). It is proposed that the specific epidermal cell fate is a consequence of WER activity being concentrated in the cells in the future N position, whereas CPC activity is present only in cells in the future H position (Schiefelbein, 2000). When the WER protein binds to TTG1/GL3/EGL3, the root hair-inhibitory complex becomes active and promotes the expression of homeodomain transcription factor GLABRA2 (GL2) (Rerie et al., 1994; Lee and Schiefelbein, 1999). In addition, GL2 transcription was shown to also depend on HDA6-mediated histone deacethylation (Li et al., 2015). GL2 in turn inhibits transcription of the bHLH transcription factor ROOT HAIR DEFECTIVE 6 (RHD6) (Masucci and Schiefelbein, 1994; Menand et al., 2007), which leads to the expression of atrichoblast-specific genes and thus specification of the non-hair fate (Masucci and Schiefelbein, 1996; Bruex et al., 2012). Further, a recent study showed that GL2 negatively regulates not only RHD6 but also other root-hair promoting bHLH genes, RHD6-LIKE1 (RSL1), RSL2, Lj-RHL1-LIKE1 (LRL1), and LRL2, by binding to their upstream regions (Lin et al., 2015). In H cells, CPC inactivates the inhibitory pathway through negative regulation of GL2 expression (Lee and Schiefelbein, 2002; Wada et al., 2002), and thus promotes expression of GL2 target genes and consequently root hair cell differentiation.

Additionally, in atrichoblast cells, the WER/GL3/EGL3/TTG1 complex positively regulates CPC and two other root hair fate-promoting transcription factors, TRIPTYCHON (TRY) and ENHANCER OF TRY AND CPC1 (ETC1) (Lee and Schiefelbein, 2002; Wada et al., 2002; Bernhardt et al., 2003; Kirik et al., 2004; Koshino-Kimura et al., 2005; Ryu et al., 2005; Simon et al., 2007). The latter is also dependent on HDA6-mediated histone deacethylation for transcriptional regulation (Li et al., 2015). For many years, the WER/GL3/EGL3/TTG1 has been proposed as the main regulatory complex of cell-fate determination. In trichomes, however, besides the well-known GL1/GL3/TTG1 trimeric complex, GL3/TTG1 and GL3/GL1 form separate dimeric complexes that differentially regulate downstream genes (Pesch et al., 2015). As such, TRY is activated by GL3/TTG1, which is counteracted by GL1. CPC is activated by GL1/GL3, which is inhibited by TTG1. The question arises if this process of alternative complex formation is also present in root hair development? Can WER for instance form a complex with GL3, activating CPC? Or does WER counteract TRY activation by GL3/TTG1?

The current understanding is that, upon activation by the WER/GL3/EGL3/TTG1 complex, CPC, TRY, and ETC1 migrate to the adjacent root hair cells and competitively inhibit binding of WER to GL3/EGL3 (Lee and Schiefelbein, 2002; Kurata et al., 2005; Kang et al., 2013), which represents a lateral inhibition mechanism (LIM; Schiefelbein et al., 2014). In addition, TRY positively regulates the expression of the leucine rich repeat receptor-like kinase SCRAMBLED (SCM; Kwak and Schiefelbein, 2014), which is thought to mediate the positional signaling between the epidermis and the cortex through negative regulation of WER transcription (Kwak and Schiefelbein, 2007). SCM is part of an autoregulatory feedback loop, where its preferential accumulation in H-cells is positively regulated by the downstream transcription factor TRY (in H-cells), and negatively regulated by the transcriptional WER/GL3/EGL3/TTG1 complex (in N-cells; Kwak and Schiefelbein, 2008). Interestingly, the kinase domain of SCM is enzymatically inactive (Chevalier et al., 2005); however, it is required for establishment of proper root hair patterning (Kwak et al., 2014). Until now, the ligand(s) of SCM remain unknown but potential downstream targets have been identified (Fulton et al., 2009; Bai et al., 2013; Trehin et al., 2013). Moreover, a zinc-finger protein called JACKDAW (JKD) has been proposed as being an upstream component of the SCM-dependent root hair regulatory network (Hassan et al., 2010).

This description demonstrates that the network of root epidermal cell patterning is highly complex and employs, besides regulatory mechanisms such as lateral inhibition, several feedback loops and interactions (reviewed in Schiefelbein et al., 2014).

Besides the abovementioned complex genetic cascades, also hormones, in particular ethylene, auxin and brassinosteroids (BRs), influence root epidermal cell fate determination. Pharmacological studies have shown that treatment with aminoethoxyvinylglycine (AVG), an ethylene biosynthesis inhibitor, or Ag^+^, an ethylene action inhibitor, abolishes root hair formation (Masucci and Schiefelbein, 1994; Tanimoto et al., 1995). Alternatively, increasing concentrations of 1-aminocyclopropane-1-carboxylic acid (ACC), a precursor of ethylene, lead to progressively more ectopic root hairs (Tanimoto et al., 1995). In addition, the recessive mutation in CONSTITUTIVE TRIPLE RESPONSE1 (CTR1), which encodes a Raf-like protein kinase that negatively regulates ethylene signaling (Kieber et al., 1993) by binding to the ethylene receptor ETR1 (Huang et al., 2003), causes development of ectopic root hairs (Dolan et al., 1994). In contrast to ethylene, application of exogenous auxins, indole-3-acetic acid (IAA), and 2,4-Dichlorophenoxyacetic acid (2,4-D), seems to have no effect on epidermal cell fate determination as no ectopic root hairs are formed (Masucci and Schiefelbein, 1996; Pitts et al., 1998) even though auxin stimulates ethylene production in roots (Abeles et al., 1992). On the other hand, both ACC and IAA are able to restore root hair formation in the root hairless *rhd6* mutant plants. Transcriptome analysis has shown that auxin and ethylene have an overlapping effect on the majority of RHD6-regulated genes, most likely due to linkage between their biosynthetic pathways (Bruex et al., 2012). RHD6 itself has, however, long been thought to function upstream of auxin signaling, and as such, the role of auxin in epidermal patterning has remained largely uncharacterized. Recently, Yi et al. (2010) showed that IAA-mediated reversion of the *rhd6* phenotype is due to activation of the bHLH transcription factor ROOT HAIR DEFECTIVE 6-LIKE 4 (RSL4). RHD6 directly targets RSL4, which is now thought to form the first auxin-responsive component in the root hair signaling pathway. RSL4 protein starts accumulating 2 h before root hair initiation and reaches a maximum during the early stages of root hair development, after which it is slowly degraded by the 26S proteasome during tip growth (Datta et al., 2015). Not only does RNAi-mediated silencing of RSL4 result in shorter root hairs, it also results in less root hairs per cell file. In addition, RSL2, the closest relative to RSL4, is also regulated by RHD6 and auxin, and the *rsl2-1 rsl4-1* double loss-of-function mutant is hairless (Yi et al., 2010). Based on these findings we can conclude that it would be worthwhile to further investigate the role of RSL4 and RSL2 in root hair initiation.

Hormone or hormone-inhibitor treatments often lead to root hairless or ectopic root hair phenotypes. In addition, interference with normal auxin signaling also results in strongly reduced root hair formation. For instance, stabilized dominant AUX/IAA proteins (and corresponding mutants, e.g., *axr2* and *axr3*) do not form root hairs (Wilson et al., 1990; Leyser et al., 1996; Knox et al., 2003). Curiously, however, no cell fate determination genes upstream of RSL4 have yet been shown to be direct targets of auxin or ethylene signaling. For sure, GL2 expression is not regulated by auxin or ethylene (Masucci and Schiefelbein, 1996). Using databases such as the PLACE database (Higo et al., 1999) a search for auxin (ARFAT; Ulmasov et al., 1995) and ethylene (ERELEE4 and GCC-box; Ohme-Takagi and Shinshi, 1990; Itzhaki et al., 1994) response elements in the promotor regions of early root hair cell fate determination genes could be conducted. The conserved ARFAT sequence (TGTCTC) is known to be a specific target for auxin-response factors (ARFs; Ulmasov et al., 1995, 1997, 1999a,b), which regulate auxin-mediated transcription after being derepressed through removal of the inhibiting interacting Aux/IAA proteins (reviewed in Rogg and Bartel, 2001). This analysis could enlighten the potentially heavily underestimated role of auxin- and ethylene-signaling in epidermal cell fate determination. In addition, an in detail analysis of the temporal transcriptional response of these genes to auxin is still lacking, but could provide valuable information.

For long, BRs have been considered to take part in regulating root hair tip growth, rather than in the preceding root epidermal cell fate determination (Kim et al., 2006). However, recent publications have shown otherwise (Kuppusamy et al., 2009; Cheng et al., 2014). Treatment of roots with brassinolide was shown to induce transcription of WER and its downstream target GL2 (Kuppusamy et al., 2009). Contrastingly, Kuppusamy et al. (2009) found no effect on the spatial distribution of GL2 transcription upon application of exogenous epibrassinolide, but Cheng et al. (2014) reported ectopic GL2 expression in H-cells in plants overexpressing the BR-receptor BRASSINOSTEROID INSENSITIVE (BRI1). Strikingly, however, Kuppusamy et al. (2009) showed that the *bri1*-mutant and brassinazole (BR biosynthetic inhibitor) treated plants had reduced WER and GL2 transcription, showed aberrant patterning of WER and GL2 expression in the epidermis, and that they contained an increase of non-hair cells in the H-position. The ectopic expression of WER and GL2 in *bri1* roots was shown to be due to decreased expression of CPC (Kuppusamy et al., 2009). A model was proposed where BR-activated BRI1 induces WER expression in future N-cells, resulting in CPC accumulation. Intercellular CPC translocation would inhibit WER and GL2 transcription in neighboring cells, and subsequently promote SCM accumulation. SCM in turn further represses WER transcription and promotes the H-cell fate. As such, positional information for root epidermal cells is conveyed through both BRI1 and SCM (Kwak and Schiefelbein, 2007; Kuppusamy et al., 2009).

The consensus states that brassinosteroids are perceived at the plasma membrane localized BRI1 receptor (Li and Chory, 1997). BRI1-induced signal transduction in turn leads to inactivation of BRASSINOSTEROID INSENSITIVE 2 (BIN2), a GSK3-like kinase which, in its inactive form, accumulates in the nucleus to regulate gene transcription (Yang et al., 2011). Interestingly, Cheng *et al.* (2014) showed that BIN2 also directly binds to WER, EGL3, and TTG1 and phosphorylates the latter two. TTG1 phosphorylation inhibits WER/GL3/EGL3/TTG1 complex activity and subsequently GL2 transcription, whereas phosphorylation of EGL3 (which is transcribed in H-cells only) was shown to be responsible for both intra- and intercellular (H- to N-cells) EGL3 transport together with GL3 (Bernhardt et al., 2005; Cheng et al., 2014).

It is now clear that BRs are important components of the epidermal cell fate determination pathway, where they promote the N-cell fate in the root epidermis. However, more evidence is needed to include these findings in the model of cell fate determination, especially regarding the spatial and temporal dynamics of BR perception and sensitivity, and subsequent cell-specific signal transduction. Is the sensitivity to BRs different in H- and N-cells? BRI1 is known to be ubiquitously expressed in the root (Friedrichsen et al., 2000), but artificial constriction of BRI1 expression in either N- or H-cells differentially affects root cell elongation (Fridman et al., 2014). Curiously, no data is available comparing BRI1 density at the plasma membrane in N- and H-cells. As such, a detailed spatial and temporal characterization of BRI1 and its downstream targets in *Arabidopsis* roots could provide useful insights.

## Switching from Diffuse to Polar Growth at a Specific Site of the Trichoblast

**FIGURE 3:**
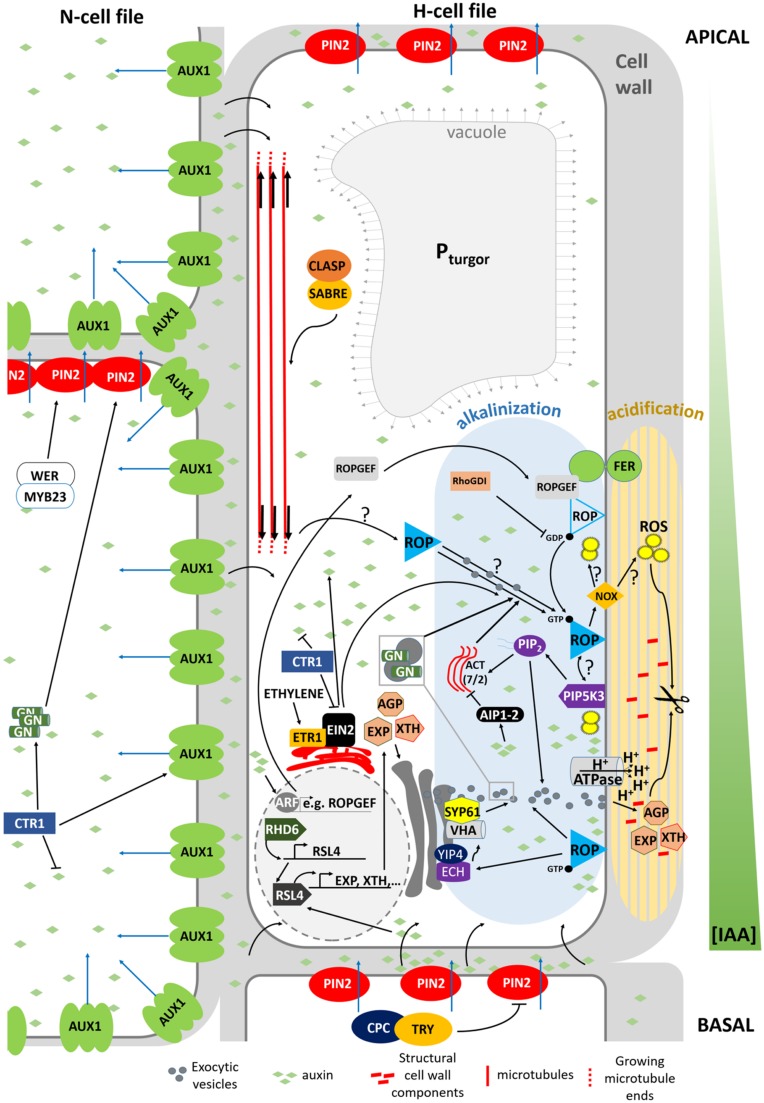
**Model summarizing the mechanism determining planar polarity within the *Arabidopsis* trichoblast.** FER, FERONIA; GN, GNOM; ACT, actin; VHA, VHA-A1; NOX, NADPH OXIDASE, ROP, ROP GTPase. Blue arrows indicate active auxin transport. Black arrows crossing the PM refer to passive diffusion of auxin.

The decision to initiate a root hair is made by the abovementioned mechanism(s) and promotes local cell wall outgrowths. But, how does a cell wall that has just adapted the architecture to prevent expansion at the end of the elongation zone, accommodate polar expansion (**Figure 3**)?

In *Arabidopsis*, root hair initiation begins with the establishment of polarity within epidermal cells and subsequent selection of the initiation site at the more basal part of the trichoblast (Carol and Dolan, 2002). Before a root hair emerges, plant RHO GTPase (RAC/ROP) proteins become polarly localized in the plasma membrane at the future growth site. These small GTPases remain present in the apical part of root hair bulges and at root hair tips until final cessation of growth, indicating that they are required for polar growth (Molendijk et al., 2001; Jones et al., 2002). This is further strengthened by the finding that mutations in ROP-interacting proteins, such as RHOGDI1/SCN1, result in a mis-localization of ROPs or in the appearance of additional ROP-spots on trichoblasts, which in turn results in altered or additional root hair initiation sites (Carol et al., 2005). In addition to polar ROP accumulation, the cell wall acidifies very locally at the site of future root hair bulge formation, whereas the cytosol becomes more alkaline. Rising external pH with buffers reversibly stops the bulge initiation process and indicates that wall acidification plays a role in the activation of cell wall loosening proteins, like expansins (Bibikova et al., 1998; Cho and Cosgrove, 2002) and xyloglucan endotransglucosylase/hydrolases (XTHs; Vissenberg et al., 2001), whose role is to re-loosen walls that just adopted an architecture to prevent further expansion. Although no loss-of-function mutants in XTH or expansin with root hair defects are described, the involvement of both proteins remains an attractive hypothesis. The expression of two *Arabidopsis* expansin genes (AtEXP7 and AtEXP18) is tightly linked to the early events of root hair formation and occurs before the cell wall bulges out (Cho and Cosgrove, 2002). The auxin-inducible EXPA7 is one of 83 genes regulated by RSL4, thereby strengthening the hypothesis that RSL4 could be a direct transcriptional regulator of root epidermal bulge formation (Yi et al., 2010). The role of expansins in root hair initiation is substantiated in Maize and Barley (Baluška et al., 2000; Kwasniewski and Szarejko, 2006). Similarly, the very localized endo-transglucosylase (XET) activity of XTH proteins occurs before visible bulge formation (Vissenberg et al., 2001). The fact that localized cell wall loosening is one of the key factors in the bulge formation is strengthened by the finding that in *rhd6* mutants crossed with *prc1-1* (mutated in the cellulose synthase *CESA6* gene resulting in reduced apoplastic cellulose; Fagard et al., 2000) the requirement for RHD6 during hair initiation is reduced. This might result from a weaker cell wall structure, due to the reduced cellulose content, which mimicks the effect of the cell wall loosening events by expansin and XTHs during the early stages of hair formation (Singh et al., 2008). Besides site-specific loosening of trichoblast cell walls, local wall compositional changes may aid normal expansion, as evidenced by the *root epidermal bulger* (*reb1/rhd1*) mutant that contains abnormally expanding trichoblast cells (Baskin et al., 1992), accompanied with changes in arabinogalactan (AGP) protein content in the walls (Andème-Onzighi et al., 2002). This all suggests that the bulge formation itself is mostly the result of local cell wall loosening and turgor-driven expansion of this apoplast site.

**FIGURE 4:**
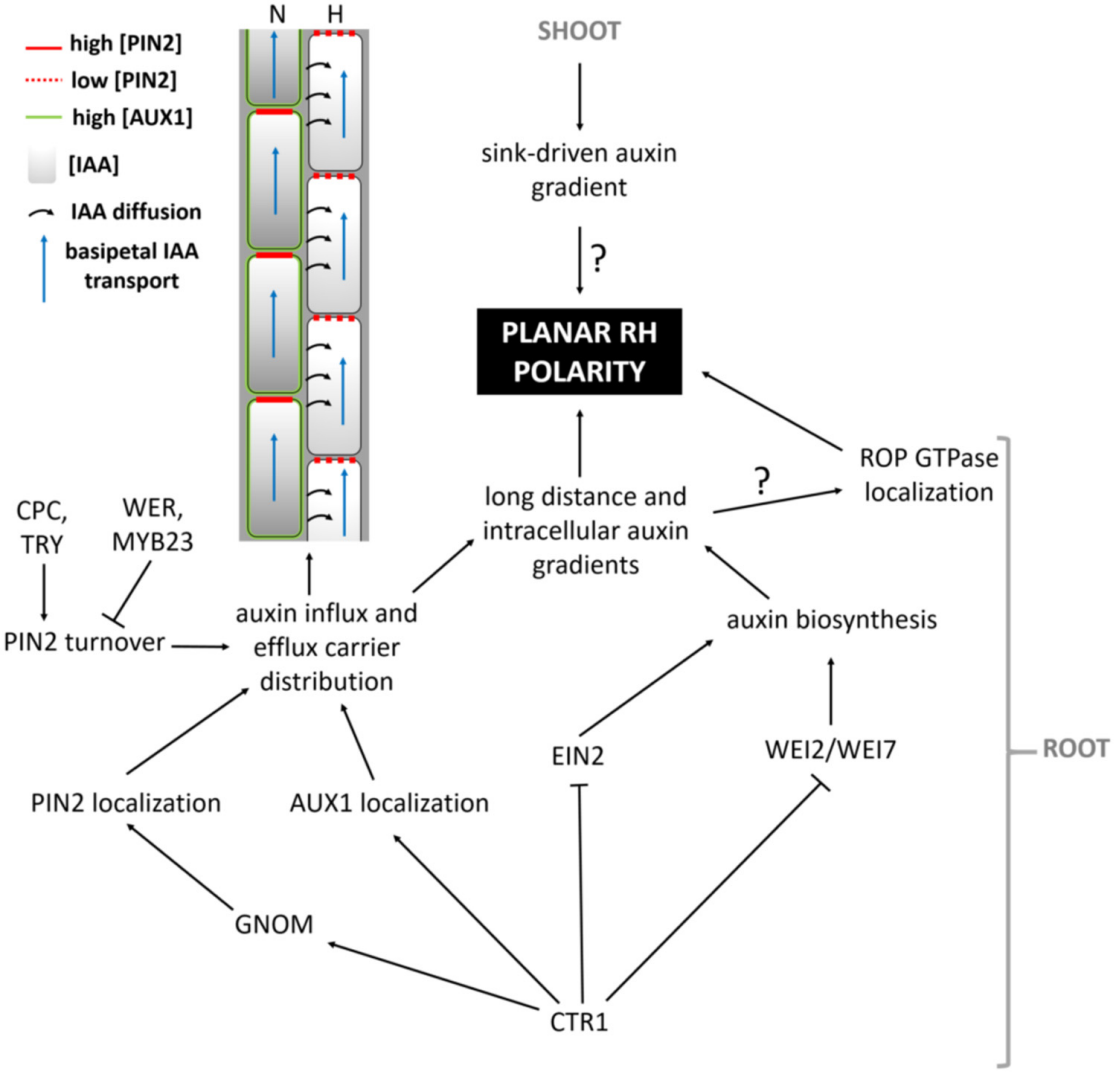
**Model summarizing the pathway leading up to the establishment of auxin-mediated planar polarity**.

Which mechanisms then determine the exact site of apoplastic acidification and cell wall loosening? Evidence comes from *axr2-1* and *rhd6*-mutants that exhibit hairs at a more shootward position of trichoblasts, and the mislocation of root hairs in the *rhd6* mutant can be rescued by either auxin or addition of an ethylene precursor (Masucci and Schiefelbein, 1994). The involvement of both hormones in site determination is further supported by mislocated hairs on the *etr* and *eto* ethylene mutants (Masucci and Schiefelbein, 1996). In addition, mutant analysis reveals that auxin may provide positional information for ROP positioning at least through activity of AUX1, EIN2, and GNOM (Fischer et al., 2006) so that hairs are formed at this site of the cell where an auxin maximum can be found (Sabatini et al., 1999; Grebe et al., 2002; Ikeda et al., 2009). So how are these auxin gradients formed, and what is their role in the establishment of planar polarity? Recent findings point toward an elegant interplay between localized regulation of auxin biosynthesis, and differential distribution of auxin in- and eﬄux carriers in N- and H-cells (**Figure 4**; Ikeda et al., 2009; Jones et al., 2009; Löfke et al., 2015). CTR1, which was previously highlighted for its role in cell-fate determination, also appears to function as a key regulator of auxin-induced planar polarity in root hair initiation (Ikeda et al., 2009). CTR1 inhibits auxin biosynthesis in a concentration-dependent manner through negative regulation of WEI2, WEI7 (key genes involved in auxin biosynthesis) and EIN2. Moreover, it is needed for uniform AUX1 localization to the plasma membrane of N-cells and, through positive regulation of GNOM, for PIN2 localization to the apical membrane of N- and (to a lesser extent) H-cells. The result is a long distance auxin gradient originating in the root tip (Ikeda et al., 2009), and intracellular auxin gradients in N-cells (high auxin concentration) and H-cells (10-fold lower auxin concentration; Jones et al., 2009). Interestingly, differential AUX1 and PIN2 accumulation/localization in N-cells (uniform AUX1 distribution and high PIN2 abundance) and H-cells (no AUX1 and 30% lower PIN2 abundance) is at least in part dependent on early cell fate determination genes (Löfke et al., 2015). In N-cells, WER/MYB23 negatively regulate PIN2 turnover, whereas in H-cells, CPC/TRY positively affect PIN2 turnover and vacuolar degradation rates. How these gradients relate to localized accumulation of ROP GTPases at the future hair site remains elusive. The link between auxin concentration and bulge site determination was described in a mathematical model that can predict localized patches of active ROPs with the assumption that ROP activity is dependent on auxin concentration (Payne and Grierson, 2009). An auxin sensing system was proposed to control ROP GTPase during cell expansion in *Arabidopsis* leaves, making that the assumption will be close to reality (Chen and Yang, 2014). Although the appearance of the F-actin cytoskeleton was not significantly different in the basal region of trichoblasts when compared with the apical ends of the same cells (Kiefer et al., 2014) and although prevention of targeted vesicle delivery by disrupting the F-actin cytoskeleton did not affect the bulge formation (Čiamporová et al., 2002), Kiefer et al. (2014) nevertheless provide evidence for the requirement of ACTIN7 (ACT7) interaction with the negative modulator ACTIN-INTERACTING PROTEIN1-2 (AIP1-2) during polar ROP placement. Furthermore, AIP1-2 expression is enriched in hair cell files, it is under control of WER and is sensitive to ethylene and auxin treatment, which makes it a modulator of auxin-gradient dependent cell polarization by ACT7. Exactly how ROP placement is achieved by ACT7 and AIP1-2 remains unclear, since early polar ROP localisation is not affected by short-term F-actin destabilization (Molendijk et al., 2001), suggesting that it happens in an indirect manner. Because Brefeldin A (BFA) inhibits the enzyme activity of *Arabidopsis* GNOM Arf GDP/GTP exchange factors (GEF) that control cycling between GTP-bound (on) and GDP-bound (off) states of ROPs and the early localization of ROP at the plasmamembrane of the future initiation site (Molendijk et al., 2001), it is possible that root hair initiation begins with an Arf-dependent, actin-independent vesicle trafficking, such as secretion at an internal cue, and is then followed by polar localization of ROP. Also the microtubular cytoskeleton helps to define the root hair initiation site. A reoriented, bipolar arrangement of longitudinally growing microtubules was detected in elongating trichoblasts just before they initiated a hair (Pietra et al., 2013). In these cells the majority of microtubule plus ends grew with apical directionality at apical ends of cells and with basal directionality at basal ends of cells, resembling what was described in elongating hypocotyl cells (Sambade et al., 2012). SABER and CLASP proteins interact and seem required to form this specific microtubular arragement, allowing correct root hair initation site determination (Pietra et al., 2013). How exactly microtubules mediate root hair initiation site determination remains unclear at the moment.

From the above, it seems ROP localisation and activity are the key determinants of root hair bulge site determination, but what are ROP’s functions and what happens downstream of ROP activity? In general, Rho GTPases are molecular switches that control a wide variety of signal transduction pathways (Etienne-Manneville and Hall, 2002), and members of one clade, plant RAC/ROPs, play important roles in regulating cell growth and polarity establishment, responses toward hormones and stress, development, reproduction, and interactions with the environment (Nibau et al., 2006; Yang and Fu, 2007; Kost, 2008). Compelling evidence suggests that for instance active ROP2 can activate NADPH-oxidases that produce ROS, which controls root hair development by altering the cell wall architecture, intracellular signaling and membrane trafficking (Foreman et al., 2003; Carol et al., 2005; Jones et al., 2007; Duan et al., 2010; Boisson-Dernier et al., 2013). Interestingly, the Receptor-Like Kinase (CrRLK) FERONIA (FER) was co-immunoprecipitated with ROP2 in a guanine nucleotide-regulated manner (Duan et al., 2010). *Fer*-mutants have collapsed, burst, to short root hairs, but unfortunately no information is available on the exact location of the root hairs on the individual cells (Duan et al., 2010). Yet, the combination of ROP2 being polarly localized and the homogeneous presence of FER in epidermal cell membranes, provides the opportunity for localized ROP2-FER interaction and related downstream effects during root hair initiation site determination. This remains, however, to be proven.

Kusano et al. (2008) provide evidence that Phosphatidylinositol Phosphate 5-Kinase 3 (PIP5K3), a key enzyme for the production of Phosphatidylinositol 4,5-bisphosphate [PtdIns(4,5)P_2_] (Oude Weernink et al., 2004), a well-studied signaling phospholipid, localizes not only to the elongating root hair apex, but also to the site of future root hair initiation. Since PIP5K3-YFP localized to growing root hairs before actual growth occurred and its fluorescence signal disappeared from the root hair tip before the root hairs elongation was complete, Kusano et al. (2008) conclude that PIP5K3 is one of the factors leading to cell expansion rather than being a result of cell expansion. The strongly reminiscent localization of ROP and PIP5K3 during root hair initiation and growth might reflect the recruitment and/or regulation of PIP5K3 by ROPs. It has been described before that the product of PIP5K3 activity, PtdIns(4,5)P_2_, modulates the functions of a variety of actin regulatory proteins and regulators of the exocytotic machinery on the plasma membrane by directly interacting with its protein targets, and also acts as a substrate for the production of secondary messengers (see references in Kusano et al., 2008; Boss and Im, 2012; Krishnamoorthy et al., 2014). Localized secretion of cell wall modifying enzymes, cell wall components or proton-ATPases could then locally acidify the apoplast, since this acidification may be due to local changes in ionic (polymer) composition of the apoplast, or to ATP-driven outward pumping of protons across an intact plasmamembrane (Bibikova et al., 1998). One line of evidence could be given by the *echidna* and *yip* mutants (Gendre et al., 2011, 2013; Boutté et al., 2013), which show impaired post-Golgi network trafficking if they fail to produce bulges. This, however, is not proven yet. In the *echidna*-mutant the localization of ROP proteins appears to be normal indicating that ECH acts downstream of ROP localization and suggesting that deposition of certain vesicle cargos are required for root hair bulge formation. Moreover, recently ECHIDNA was shown to regulate the secretion of cell wall polysaccharides through interaction with the YPT/RAB GTPase interacting proteins 4a and 4b (YIP4a, YIP4b) and regulation of TGN components VHA-a1 and SYP61 (Gendre et al., 2013). A second and more direct line of evidence for localized secretion of certain components during root hair initiation is given by the tip-accumulation of RabA1d, labeling trans-Golgi network vesicles, in the root hair bulges (Berson et al., 2014). Taken together, whether active ROP alone or in combination with FER activates the formation of ROS and PtdIns(4,5)P_2_ to start the apoplastic acidification and further wall loosening remains to be solved. The whole cascade is summarized in **Figure 3**.

## Tip Growth Maintenance and Termination

**FIGURE 5:**
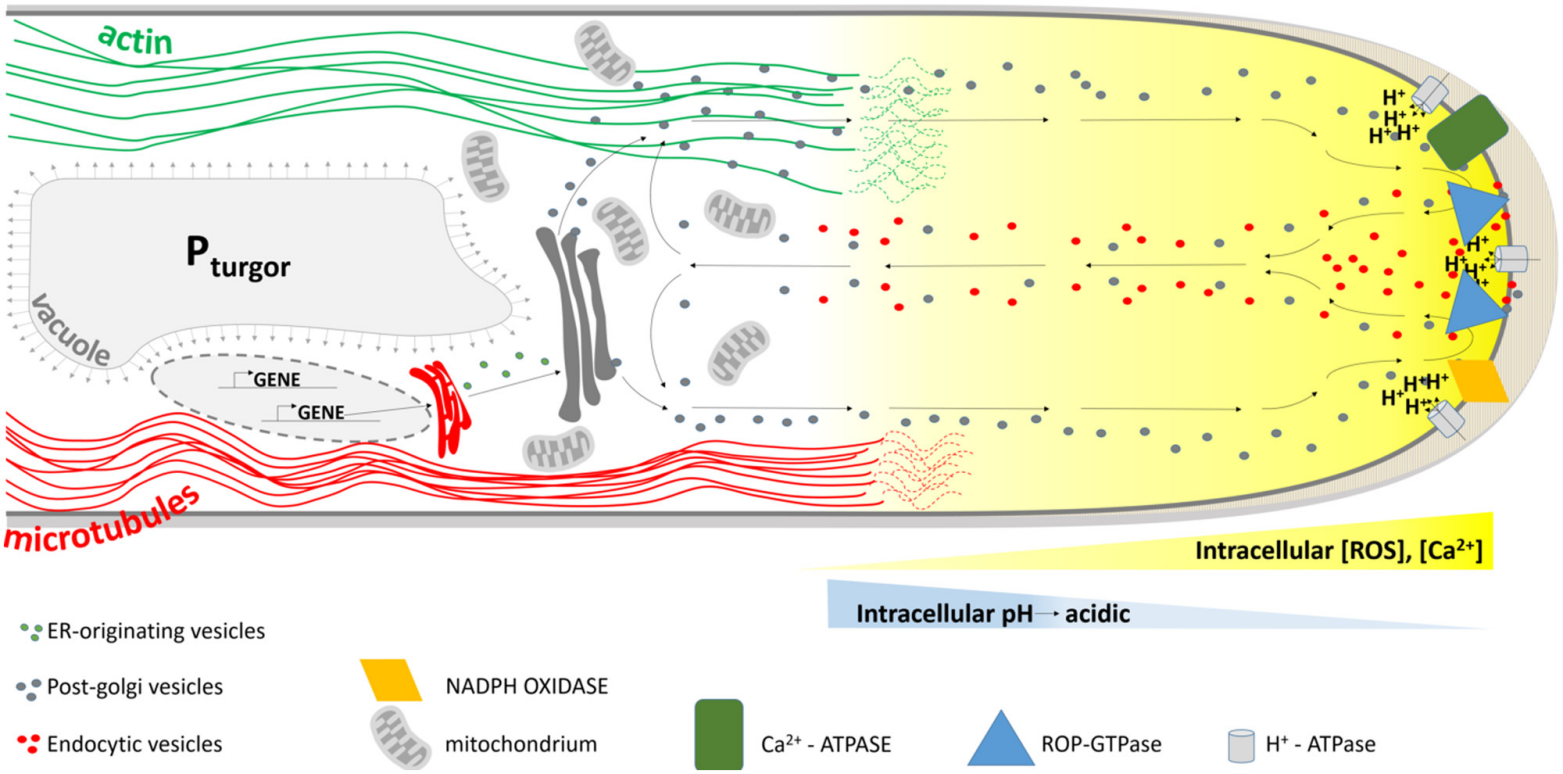
**Schematic representation of the cytoarchitecture within the growing root hair apex.** The tip is packed with membrane-bound vesicles originating from the ER and Golgi apparatus delivering new cell wall material to the growing tip. Together with endocytotic vesicles that are formed at the extreme tip they display a reverse fountain streaming. ROP proteins are predominantly localized to the tip, together with hyperpolarization-activated Ca^2+^-ATPases and NADPH oxidases. The latter are responsible for the formation of a tip-focused calcium and ROS gradient (yellow gradient). A tip-focused pH gradient is also present. Microtubules (red lines) run along the length of the hair and control the hair’s growth direction, whereas actin cables (green) allow for polar vesicle trafficking.

When the bulge is fully formed, the transition from initiation to tip growth begins with the accumulation of secretory vesicles at the apical part of the bulge. A tip growing root hair has a highly organized cytoarchitecture (**Figure 5**). The hemispherical apex is filled with densely packed vesicles while small organelles such as Golgi stacks, mitochondria, endoplasmic reticulum and plastids are present in a sub-apical region. The more basal part of a hair contains a large vacuole that occupies most of the space. The nucleus enters the hair and follows the growing tip at a constant distance. When a root hair becomes mature and growth ceases, this highly polar organization of the cytoplasm disappears (Ryan et al., 2001). Several players are known that mediate the tip growth of root hairs, but fall out of the scope of this review.

## Concluding Remarks

Abundant genetic, molecular, and mutant resources have made that the *Arabidopsis thaliana* root is being successfully exploited as a model system to elucidate complex molecular pathways leading to cell fate determination, planar cell polarity, and tip growth. Many players in these pathways have been described. However, undoubtedly several are yet to be identified. The influence and action of phytohormones such as auxin, ethylene and brassinosteroids on root architecture and development is generally recognized, yet their roles in the different developmental processes are far from fully understood. Several open questions thus still remain, and answers are needed to understand the intriguing complexity of development on the single cell level.

## Author Contributions

DB, SS, and KV studied the literature, critically reflected on the current knowledge and wrote the review.

## Conflict of Interest Statement

The authors declare that the research was conducted in the absence of any commercial or financial relationships that could be construed as a potential conflict of interest.
